# Influence of time and hydration (ageing) on flexural strength of Yttrium stabilized Zirconia polycrystals (Y-TZP) fabricated with different CAD-CAM Systems

**DOI:** 10.12669/pjms.37.3.3996

**Published:** 2021

**Authors:** Khulud A Al-Aali, Saad Alresayes, Aasem M Alhenaki, Fahim Vohra, Tariq Abduljabbar

**Affiliations:** 1 Khulud A. Al Aali, Department of Clinical Dental Sciences, College of Dentistry, Princess Nourah Bint Abdulrahman University, Riyadh, Saudi Arabia; 2 Saad Alresayes, Department of Prosthetic Dental Sciences, College of Dentistry, King Saud University, Riyadh, Saudi Arabia; 3 Aasem M Alhenaki, Department of Prosthetic Dental Sciences, College of Dentistry, King Saud University, Riyadh, Saudi Arabia; 4 Fahim Vohra, Department of Prosthetic Dental Sciences, Research Chair for Biological Research in Dental Health**,** College of Dentistry, King Saud University, Riyadh, Saudi Arabia; 5 Tariq Abduljabbar. Department of Prosthetic Dental Sciences, Research Chair for Biological Research in Dental Health, College of Dentistry, King Saud University, Riyadh, Saudi Arabia

**Keywords:** Flexural strength, Ageing, Low temperature degradation, Zirconium oxide

## Abstract

**Objectives:**

To evaluate the effect of time and hydration (ageing) on flexural strength of yttrium-stabilized zirconia polycrystals (Y-TZP) zirconia fabricated from three different materials.

**Methods:**

This in-vitro study was performed from June to September 2019. Y-TZP bars, measuring 2 x 3 x 20 mm were prepared and sintered from three different materials, Group-1: LAVA™ Zirconia (3M ESPE, US) (control) Group-2: Vita In-Ceram YZ (VITA, Germany) and Group-3: Aadva™ Zirconia (Zr) (GC Advanced technologies Inc.). 30 zirconia bars per group were prepared using sectioning of blocks with isomet saw. Followed by sintering in furnaces for recommended temperature cycles. One side of bars were polished and beveled for flexural testing. Groups of specimens were divided into subgroups of 3 (n=10) based on the ageing (distilled water in the incubator at 37ºC) durations (48 Hrs and two and half years). Ten specimens in each material groups were not aged (controls). Samples were exposed to a static force in a three-point bend test using a universal instron-testing machine until fracture. Scanning electron microscopic assessment was performed for fractured specimens for ageing. Data was analyzed using ANOVA and Tukey post hoc test.

**Results:**

The mean flexural strength at baseline for Group-1: LAVA™ Zirconia, group (632.7 ± 136.5 MPa) 2: Vita In-Ceram YZ (1036.3 ± 229.6 MPa), and Group-3: Aadva™ Zirconia (1171.3 ± 266.3 MPa) were significantly different. Group-2 and Group-3 specimens showed higher strength compared to Group-1 specimens, irrespective of the ageing duration (p<0.05). Analysis of pooled data for flexural strength for materials by aging period (baseline, after 48 hours and after 2 and ½ years) showed that there was significant reduction of strength with increasing duration (p<0.05).

**Conclusions:**

Y-TZP showed variations in flexural strength depending on the material type. Ageing duration exhibited significant influence on the flexural strength of Y-TZP when comparing no ageing to two and half years. Vita In-Ceram YZ and Aadva Zirconia (Zr) showed higher and clinically acceptable flexural strength outcomes.

## INTRODUCTION

Yttrium stabilized zirconia polycrystals (Y-TZP) is commonly used in oral rehabilitation as fixed partial dentures for replacement of posterior teeth. Y-TZP ceramics have several advantages over other ceramic materials, due to the transformation toughening mechanism that allows it to reinforce mechanical properties. However, a common post-operative complication for the bilayered Y-TZP FPDs is veneering ceramic chipping and fracture.^1^,^2^ Among the causes for veneer fractures is the compromise in flexural strength of Y-TZP frameworks due to intra-oral masticatory fatigue and ageing. When occlusal forces are applied directly through the long axis of a Y-TZP fixed partial denture at the pontic, compressive stresses develop at the occlusal aspect of the connector at the marginal ridge, and tensile stresses develop at the gingival surface of the connector.^3^

Y-TZP mechanical degradation is known as ‘aging’, and is due to the progressive spontaneous transformation of the metastable tetragonal phase into the monoclinic phase. This behavior is observed in the temperature range above 200°C in the presence of water vapor.^4^,^5^ In addition, Low temperature degradation (LTD) includes the transformation of stabilized tetragonal zirconia to monoclinic phase at the surface of the specimen in the presence of water at relatively low temperatures.^6^ Aging of zirconia can have detrimental effects on its mechanical properties. And mechanical stresses and wetness exposure are associated with the acceleration of this process.^7^ It is suggested that ageing results in a reduction in strength, toughness and density, and an increase in monoclinic phase content.^7^ The transformation from Tetragonal to Monoclinic phase starts on the surface and progresses into the material bulk, enhanced in the presence of water or vapor. Moreover, reduction in grain size and increase in concentration of stabilizing oxide reduces the transformation rate.^7^

In a study by Swab et al., ten materials were investigated for mechanical strength in presence of water vapor at low temperature.^7^ It was concluded that different levels of strength degradation occurred in all the materials except one, where strength remained the same after the treatment.^7^ These findings were attributed to the ageing behavior and how it is related to the differences in equilibrium of microstructural parameters e.g. yttria concentration and distribution, grain size, flaw population and distribution in the samples tested.^6^ In a similar study, use of gamma sterilization or aging in Ringer’s solution for 100 days did not induce significant variations in the strength of Y-TZP samples.^8^,^9^ Therefore it appears that the type of Y-TZP material and its structural configuration may be a critical factor in their ability to resist ageing and LTD, in the presence of temperature changes, moisture and masticatory forces (oral environment).

Contemporary Y-TZP CAD-CAM systems are continuously introduced in the field of dentistry for improved development of complex dental restorations. Such newer systems prescribe their own Y-TZP materials, scanner and milling instruments for increased accuracies and compatibility. On such system is the Aadva™ Zirconia- (GC Advanced technologies) allowing for fully automated Y-TZP restoration manufacturing with super precision.^10^ To our knowledge from indexed literature there is limited comparative data of contemporary Y-TZP systems, on the influence of ageing on their mechanical properties, particularly flexural strength. It is hypothesized, that contemporary Y-TZP materials and systems in comparison to conventional systems with respect to duration and hydration (ageing) will show comparable flexural strength outcomes.

Therefore, the aim of the present study was to evaluate the effect of time and hydration (ageing) on flexural strength of Y-TZP zirconia fabricated from three different materials.

## METHODS

This in-vitro study was conducted June to September 2019 and was reported in accordance with checklist for reporting In-vitro studies (CRIS guidelines) and the methods were adopted from previous study^7^.

### Preparation of bars:

Y-TZP bars were prepared from three different materials, including, LAVA™ Zirconia- 3M™ ESPE, USA (Group-1), Vita In-Ceram YZ- VITA, Germany (Group-2) and Aadva™ Zirconia- GC Advanced technologies Inc. (Group-3). Y-TZP blocks were attached with the metal stubs to the Isomet 2000® Precision Saw, 0.5 mm (Buehler Ltd). After tightening, blocks were aligned and sectioned under water irrigation at a load of 100 grams and 400 R.P.M. Sectioning resulted in 3 x 4 x 22 mm rectangular blocks, to compensate for the cutting saw thickness and expected firing shrinkage, with a final dimension of 2 x 3 x 20 mm after sintering. A total of ninety bars were prepared, thirty per each material type.

### Sintering and polishing of bars:

**Fig.1:**
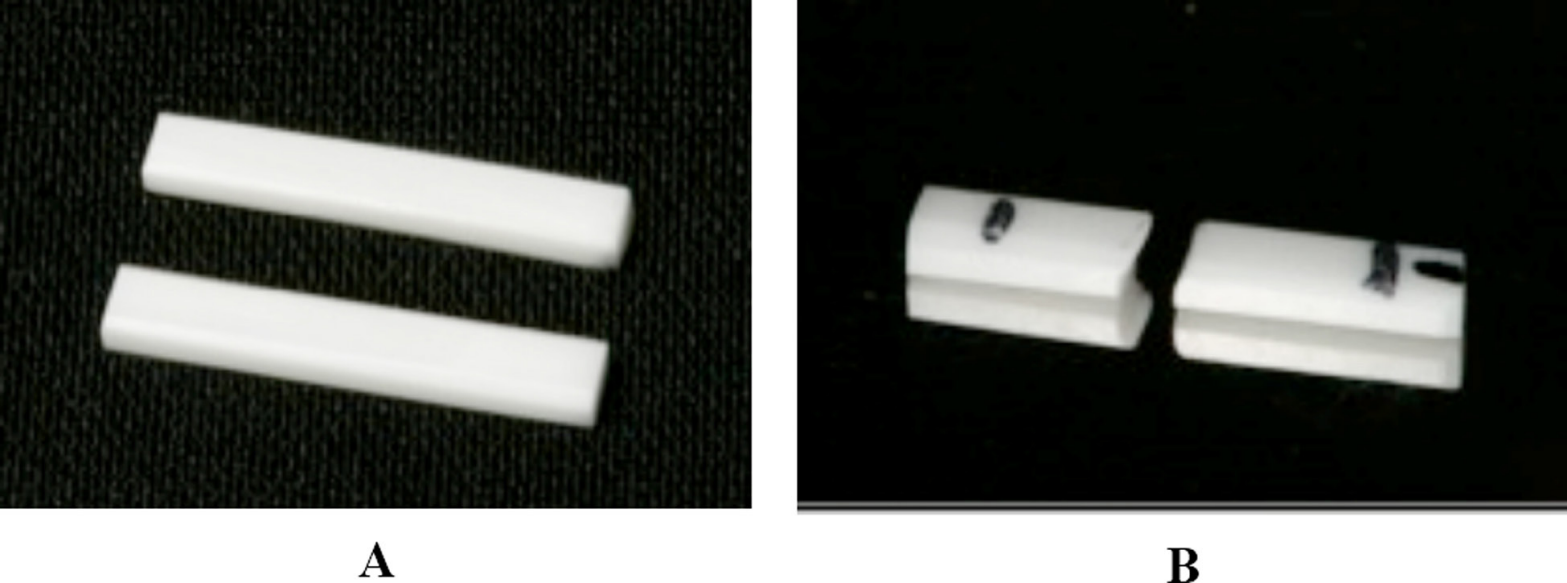
Zirconia bars after sintering (A) and bars after fracture (B).

Vita In-Ceram YZ and LAVA™ Zirconia sintering to full density was performed in the VITA ZYRCOMAT® sintering furnace (VITA Zahnfabrik) and LAVA™ furnace 200, according to manufacturer’s instructions. For the Aadvas™ Zirconia (Zr), sintering was performed in the high temperature tube furnace 59300 (Thermolyne) with air firing at 500^º^ C for 1 minute followed by an increase to 1000^º^ C at a rate of 100^º^ C/minute, and kept at 1000^º^ C for 15 minutes. All bars were polished on one side with Buehler grinding-polishing system (Buehler Ltd), starting with graded diamond grits 45 and 15 µm with water and continuing with 6-µm and 1-µm polycrystalline diamond suspension. After polishing, all zirconia bars corners were beveled with Buehler grinding-polishing system, Ecomet 3 (Isomet, Buehler Ltd). And a black mark was placed on the side of the bar, to indicate the non-polished surface (Fig.1).

All bars in each material group were further divided into three subgroups based on the duration of ageing exposure.

*Group-1 (LAVA™Zirconia). 1A:* no storage in distilled water (DW), 1B: 48 Hrs storage in DW, and 1C: Two and half years of DW storage (Control group).

*Group-2 (Vita In-Ceram YZ- VITA). 2A:* no storage in distilled water (DW), 2B: 48 Hrs storage in DW, and 2C: Two and half years of DW storage.

*Group-3 (Aadva™ Zirconia- GC). 3A:* no storage in distilled water (DW), 3B: 48 Hrs storage in DW, and 3C: Two and half years of DW storage.

Specimens for ageing were stored in distilled water filled container inside the incubator at 37º C for 48 hours and two and a half years for each group. Distilled water was checked on a regular basis to avoid dehydration of the bars.

### Three- point bend test for flexural strength:

All specimens were tested using a three-point bend test using a universal instron-testing machine (Model # 4202; Instron Corp, Canton, Mass) at a crosshead speed of 0.5 mm per minute with a 200-lb/-1kn/ 100-kg load cell. A 15 mm span 3-point fixture was used, and the bars were positioned to generate maximum tensile force on the polished surface (inferior surface) and compressive force was exerted on the superior surface of the bar. After failure “fracture”, the mean height and width of each part of the bar (Fig.1) were measured near the fracture sites using a micrometer (Model No. CD-4”CS; Mitutoyo Corp., Japan). The flexural strength (MPa) was calculated using the formula:

3 PL/2 WT2 where P is the load recorded at fracture, L is distance between supports, W is specimen width and T is the specimen thickness.^11^

### Statistical Analysis:

Means and standard deviations of flexural strengths were identified and normality was assessed using Kolmogorov Smirnov test. One-way Analysis of Variance (ANOVA) was used to detect significant differences between the groups and Tukey-Kramer multiple comparisons test was applied to detect significant differences between each groups. A p value of <0.05 were considered significant for all statistical analyses. Analysis was performed using StatPro® add-on for Microsoft Excel® software package 2011.

### Ethical Approval:

(Ref: 283/19/FR, Dated: 17-09-2019).

## RESULTS

The mean flexural strength at baseline for Group-1: LAVA™ Zirconia, Group-2: Vita In-Ceram YZ, and Group-3: Aadva™ Zirconia were 632.7 ± 136.5 MPa, 1036.3 ± 229.6 MPa, and 1171.3 ± 266.3 MPa respectively (Table-I). The specimens in Group-2 and Group-3 exhibited significantly higher flexural strength values (p<0.05) as compared to Group-1 specimens at baseline. At 48 hours of ageing (Groups-B), mean flexural strengths for Group-1, Group-2 and Group-3 were 607.5±70.5 MPa, 955.1±248.6 MPa, and 1069.1±306.4 MPa respectively. Overall, there was a decrease in flexural strength for all groups after 48 hours of ageing. Specimens exposed to 2 and ½ years of ageing (Groups-C), showed the least flexural strength values within each material group. With Group-1, Group-2 and Group-3 showing 588.5±80.5 MPa, 925.2±234.3 MPa and 944.7±123 MPa respectively.

**Table I:** Means and standard deviations of flexural strength at baseline, 48 hours and 2 and ½ years of aging.

Study groups	Baseline (Mean±SD)	48 hours (Mean±SD)	2 and ½ years (Mean±SD)	ANOVA
Group-1-LAVA Zirconia	632.7 ± 136.5^A a^	607.5 ± 70.5^A a^	588.5 ± 80.5^A^ ^a^	
Group-2-Vita In-Ceram YZ	1036.3 ± 229.6^B a^	955.1 ± 248.6^B a^	925.2 ± 234.3^B a^	p < 0.001
Group-3-Aadva Zirconia	1171.3 ± 266.3^B a^	1069.1 ± 306.4^B ab^	944.7 ± 123^B b^	

Different superscript capital alphabet in same column denotes statistically significant difference. (Tukey Post hoc test) Different superscript small alphabet in the same row shows statistical difference. (Tukey Post hoc test).

**Fig.2:**
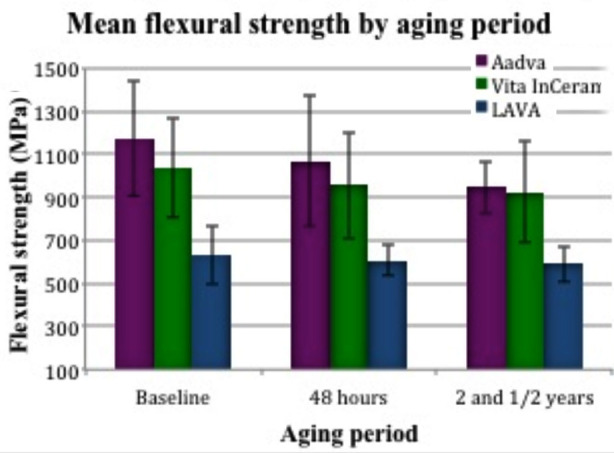
Comparison of flexural strengths (MPa) by aging period among study groups

There was no significant difference between Group-2 and Group-3 materials with aging period. However, Group-1 had the lowest flexural strength values compared to the other materials in all aging periods. Multiple comparisons test (Tukey’s post hoc test) showed that there is significant flexural strength difference (p<0.05) between Group-2 and Group-1 and between Group-1 and Group-3 specimens (p<0.05) respectively. However, there was no significant difference (p>0.05) between Group-2 and Group-3 specimens tested at baseline (Group-A), after 48 hours (Group-B) and after 2 and ½ years of aging (Group-C) in distilled water (Fig.2).

**Fig.3:**
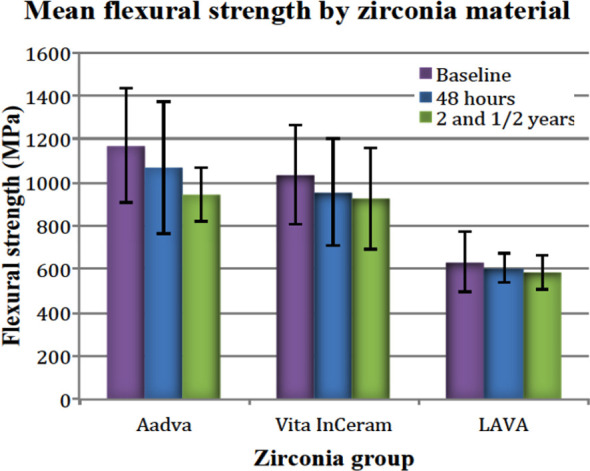
Comparison of mean flexural strengths (MPa) by material groups in the study

**Fig.4:**
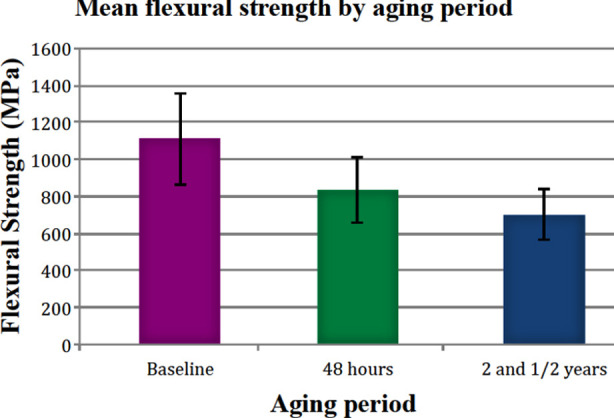
Comparison of pooled flexural strengths for all study materials by aging durations.

Comparison of flexural strength values within each material showed that there was no significant difference between the baseline (Group-A) and aging for 48 hours (Group-B) and 2 and ½ years (Group-C) in distilled water for each tested zirconia group (p>0.05), except Group-3. Group-3 materials showed a significant difference between baseline (Group-A) and after 2 and ½ (Group-C) years of aging (p<0.05). We can say that there was a diminishing behavior in flexural strength after aging in water at 37ºC as shown in Fig.3. Statistical analysis of pooled data for flexural strength for materials by aging period [baseline (Group-A), after 48 hours (Group-B) and after 2 and ½ years (Group-C)] showed that there was significant difference (p<0.05) as presented in Fig.4. The pooled flexural strengths for all materials at baseline, at 48 hours and 2 and ½ years of aging were 1108.2 ± 244.7 MPa, 832.3 ± 175.2 MPa and 698.3±136.6 MPa respectively.

For aged and un-aged zirconia surfaces influence on grain size was observed, Group-1 specimens showed increased roughness and more surface grain and pullout in comparison to the un-aged specimens.

## DISCUSSION

The present study was based on the hypothesis that contemporary Y-TZP materials and systems (Vita In-Ceram YZ- VITA and Aadva™ Zirconia- GC) in comparison to conventional systems (LAVA™ Zirconia) will show comparable flexural strength outcomes in the presence of ageing. It was observed that the type and systems of Y-TZP material had a significant effect on the flexural strength. In addition, the ageing durations had a significant but smaller negative impact on the flexural strength of the Y-TZP materials tested. Therefore, rejecting the hypothesis. Explanations for these findings are manifold, including material composition and structure, test methodology and experiment conditions, material preparation and Y-TZP transformations.

The mechanical behavior of zirconia in the oral cavity is compromised by low temperature degradation. In the presence of moisture at isolated surface grains, ageing process is initiated resulting in dissolution of zirconium oxide bonds. This results in transformation of a stable form of tetragonal Zr to monoclinic form.^5^,^12^ This degradation of Zr occurs over several years, and therefore a two and half year duration of ageing was included in the present study. Although at high temperatures the degradation can be accelerated, samples were aged at 37º C to simulate the oral environment. In addition, a three-point bending test to assess the flexural test was employed in contrast to four-point testing systems. Three-point test employs simpler test fixtures and concentrates stress to a smaller area in contrast to four-point test, which dissipates loads over a wider area. In addition, three-point test shows higher strength and are easily adapted to high temperatures and fracture toughness testing. It is difficult to ascertain the accurate contents of the Zr materials used in the study, however they contained varying contents of Alumina, 3 mol% or more of Yttria and variable grain sizes.

The observed flexural strength among the group of materials was significantly different. Group-1 showed significantly lower strength values compared to Group-2 and Group-3 specimens irrespective of the ageing process. This difference of strengths can be attributed to material grain size and yttria content. Studies have suggested that grain size and yttria content effects the retention of tetragonal phase in contrast to the formation of cubic Zr.^13^,^14^ A critical grain size corresponds to the mol% of yttria for the retention of tetragonal phase in Y-TZP. A mismatch or increase of grain size with respect to the critical grain size will reduce the metastable tetragonal phase and therefore compromise the material strength.^15^ Previous studies have shown that a replacement or presence of cubic zirconia has shown a compromise in the strength of the materials assessed.^16^ In the present study the material compositions are difficult to accurately ascertain as the manufacturers provide minimum information. Therefore, the authors hypothesize that the relative ratio of grain size and yttria content are possible explanations for the outcomes observed.

In the present study, an overall low but significant effect of ageing on Y-TZP specimens for two and half years was shown. This is related to the phase transformations explained earlier, starting from the surface and progressively towards the material bulk. The tetragonal transforms to monoclinic phase, resulting in spaces and a change in grain volume and size. A relative increase in yttria content and grain size reduction or a simultaneous occurrence, results in a Y-TZP material with resistance to low temperature degradation. This indicates that there is a behavioral decrease in flexural strength of Y-TZP with aging. Similar findings have been presented in previous studies.^15^,^17^ Therefore, to improve the resistance of Y-TZP to ageing degradation, grain growth can be manipulated with modifications in the sintering of the materials. In addition, maintaining the yttria content to 3 mol% may also improve resistance to LTD.

In in-vitro experiments, which are for short durations, low temperature ageing has not shown negative effects on flexural strength of Y-TZP materials.^18^ However, factors to which zirconia restorations are exposed in the oral environment (e.g., cyclic, mechanical and thermal loading) result in a rapid process of ageing than in-vitro conditions. The findings in the present study are in line with previous studies, in suggesting that ageing can compromise the mechanical properties of Y-TZP in the long-term.^19^ In addition, the presence of alumina in the Y-TZP contents, support the nucleation of zirconia and robust grain boundaries.^20^ Therefore augmenting the potential for resistance to ageing and improving flexural strength. Material assessed in Group-3 of the present study, has been reported to contain Alumina in varying concentrations,^21^ therefore showing higher flexural strengths compared to Group-1 and Group-2 specimens.

Interestingly, the flexural strengths observed for Y-TZP in the present study for Group-2 and Group-3 are within the requirements of ISO 6872:2015 (ISO Class 5) and are indicated for up to 3-to-4-unit FPDs.^22^ However these findings should only be applied to the materials assessed in the study. From a clinical perspective, improving the connector height and width, in addition to the heat treatments during processing, can modify Y-TZP fixed partial denture frameworks for posterior teeth. This reduces the potential tensile stresses created within the framework, due to cyclic fatigue in the oral cavity, improving the resistance to flexion and fractures.^23^ Furthermore, introduction of contemporary stabilizers and dopants to improve mechanical and esthetic outcomes of Y-TZP materials have been proposed.^24^ Therefore, further studies assessing long-term in-vivo ageing of bio-engineered contemporary Y-TZP materials are recommended for development of versatile clinically successful stabilized zirconia.

## CONCLUSIONS

Y-TZP showed variations in flexural strength depending on the CAD-CAM systems. Ageing duration exhibited significant influence on the flexural strength of Y-TZP when compared to non-aged to 2 and half years ageing. Vita In-Ceram YZ and Aadva Zirconia (Zr) showed higher and clinically acceptable flexural strength outcomes.

### Authors’ Contribution:

**KAA and SA:** Data collection, study design, manuscript writing, final manuscript approval.

**AMA:** Data collection, study design, manuscript drafting, data analysis, and manuscript approval.

**FV and TA:** Data collection, manuscript approval, data interpretation, writing, revise, and editing and final manuscript approval.

**FV** is responsible and accountable for the accuracy of the study.
